# Light-modulated vertical heterojunction phototransistors with distinct logical photocurrents

**DOI:** 10.1038/s41377-020-00406-4

**Published:** 2020-09-23

**Authors:** Jiayue Han, Meiyu He, Ming Yang, Qi Han, Fang Wang, Fang Zhong, Mengjian Xu, Qing Li, He Zhu, Chongxin Shan, Weida Hu, Xiaoqing Chen, Xinran Wang, Jun Gou, Zhiming Wu, Jun Wang

**Affiliations:** 1 School of Optoelectronic Science and Engineering, University of Electronic Science and Technology of China, Chengdu, 610054 China; 2 State Key Laboratory of Infrared Physics, Shanghai Institute of Technical Physics, Chinese Academy of Science, 500 Yutian Road, Shanghai, 200083 China; 3 Hangzhou Institute for Advanced Study, University of Chinese Academy of Sciences, Hangzhou, 310024 China; 4 Henan Key Laboratory of Diamond Optoelectronic Materials and Devices, School of Physics and Engineering, Zhengzhou University, Zhengzhou, 450001 China; 5 National Laboratory of Solid State Microstructures, School of Electronic Science and Engineering, and Collaborative Innovation Center of Advanced Microstructures, Nanjing University, Nanjing, 210093 China; 6 State Key Laboratory of Electronic Thin Films and Integrated Devices, University of Electronic Science and Technology of China, Chengdu, 610054 China

**Keywords:** Optical properties and devices, Optical sensors

## Abstract

The intriguing carrier dynamics in graphene heterojunctions have stimulated great interest in modulating the optoelectronic features to realize high-performance photodetectors. However, for most phototransistors, the photoresponse characteristics are modulated with an electrical gate or a static field. In this paper, we demonstrate a graphene/C_60_/pentacene vertical phototransistor to tune both the photoresponse time and photocurrent based on light modulation. By exploiting the power-dependent multiple states of the photocurrent, remarkable logical photocurrent switching under infrared light modulation occurs in a thick C_60_ layer (11 nm) device, which implies competition of the photogenerated carriers between graphene/C_60_ and C_60_/pentacene. Meanwhile, we observe a complete positive-negative alternating process under continuous 405 nm irradiation. Furthermore, infrared light modulation of a thin C_60_ (5 nm) device results in a photoresponsivity improvement from 3425 A/W up to 7673 A/W, and we clearly probe the primary reason for the distinct modulation results between the 5 and 11 nm C_60_ devices. In addition, the tuneable bandwidth of the infrared response from 10 to 3 × 10^3^ Hz under visible light modulation is explored. Such distinct types of optical modulation phenomena and logical photocurrent inversion characteristics pave the way for future tuneable logical photocurrent switching devices and high-performance phototransistors with vertical graphene heterojunction structures.

## Phototransistors: a carbon sandwich for logic with light

Two forms of carbon and a hydrocarbon are combined in a phototransistor, which creates changes in optical and electronic behavior in response to different wavelengths of light, with properties that will be useful for both research and commercial applications. The innovative phototransistor has been developed by Jiayue Han and colleagues at the University of Electronic Science and Technology of China, in Chengdu, with co-workers at other Chinese research centers. It has a layer composed of spherical C60 molecules, sandwiched between the form of carbon known as graphene and the hydrocarbon pentacene. Gold and silicon-based electrodes complete the structure. The optoelectronic properties created at the junctions between the different layers overcome limitations of simpler graphene based devices. The modulation of optical and electronic behavior achieved by the system could be used in new logical switching devices.

## Introduction

Graphene has promising potential in fabricating phototransistors and photodetectors owing to its unique band structure with prominent optoelectronic and charge transport properties^1–3^. Tremendous prospects have emerged in transparent devices^4,5^, silicon compatible devices^6^, and optical modulators^7–9^. However, the inferior light absorption of single atomic layer graphene limits the phototransistor performance^10^. 2D/semiconductor heterojunctions are expected to break the electron-hole occupation symmetry, giving rise to the generation of photocurrents, including in quantum dot-enhanced structures^11,12^, stacked 2D heterojunction structures^13–17^, 2D planar structures^18,19^, dual photogating phototransistors^20^, waveguide-integrated enhanced structures^21,22^, bilayer-enhanced structures^23,24^, and even nano-graphite structures^25,26^. In particular, some typical 2D localized field methods incorporating 2D, organic, perovskite, and Dirac materials were subsequently proposed to realize high-performance phototransistors^23,24,27–33^. Numerous localized field-enhanced phototransistors have been integrated with an electrostatic field to achieve distinct features such as a floating-gate structure^27,28^, a built-in field^19^, a photogating electric field^23,24,29,30^ and a ferroelectric field^31^. For example, the ferroelectric field aims to suppress the dark current, and the floating gate aims to promote the light sensitivity.

As another unprecedented external intervention, light modulation is seldom researched for tuning optoelectronic properties. A method involving 450 nm light illumination that can expand the photodetection range and suppress the dark current was proposed by Fang et al.^34^. Furthermore, Gao et al.^35^ demonstrated Schottky phototransistors with enhanced photocurrents due to a Schottky–Ohmic transformation induced by light. As we known, comprehensive modulation of the photocurrent magnitude, operating speed, and signal direction of phototransistors by modulation light input is lacking, where light modulation hopefully offers a versatile platform for replacing electrical modulation to explore more intriguing optoelectronic devices.

In this paper, we demonstrate a light modulation method to adjust the response magnitude, speed, and direction of photocurrents based on graphene/C_60_/pentacene phototransistors with different thickness C_60_. We observe a logical photocurrent reversal and photocurrent-response time dynamics (response time varies from 550 to 20 μs) driven by light modulation in a thick-intermediate-transport layer device. In addition, in the thin C_60_ (5 nm) phototransistor, we exploit an efficient method to provide a nearly two- to threefold improvement in the visible light photoresponsivity and up to a 10^3^-fold improvement in the infrared response time under suitable light modulation. In addition, we observe both positive and negative alternating photocurrents as a function of the incident light power density. This peculiar phenomenon enables us to analyze the charge-transfer (CT) process and study the potential for new functional devices in the graphene/C_60_/pentacene system. The combination of distinct mechanisms and novel light modulation extends the functions of conventional phototransistors and promotes the device performance.

## Results

**Fig. 1:**
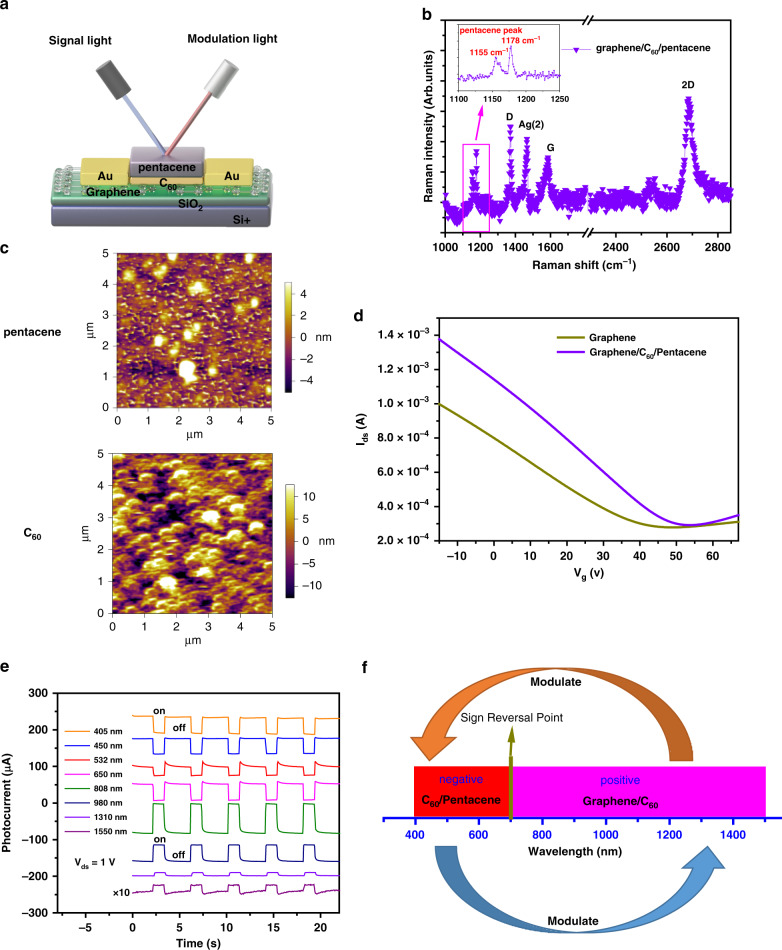
Basic characterization of the bilayer/graphene heterojunction phototransistor. **a** Light-modulation schematic diagram, and device structure diagram. **b** Raman spectrum recorded for the bilayer/graphene heterojunction phototransistor; the inset presents the pentacene characteristic peaks at 1155 cm^−1^ and 1178 cm^−1^. **c** The top panel shows a surface atomic force microscopy (AFM) image of pentacene, and the bottom panel shows a surface AFM image of C_60_. **d** *I*_ds_–*V*_g_ characteristics of the graphene phototransistor before and after deposition of C_60_/pentacene (*V*_ds_ = 200 mV). **e** Photocurrent characteristics at a source-drain bias voltage of 1 V under different wavelength radiation. **f** Schematic diagram of the opposite light doping effects to modulate the graphene channel

The light modulation transistor based on the vertical graphene/C_60_/pentacene heterojunction in this study has a typical photoconductive structure, with the schematic depicted in Fig. 1a. The bilayer heterojunction C_60_/pentacene acts as the main light-absorbing layer, which allows photogenerated free carriers to be injected into graphene (single-layer graphene is confirmed by Raman spectroscopy, Fig. 1b), modulating the conductance of the channel. C_60_/pentacene bilayer small molecule films with 5 and 10 nm thicknesses are deposited onto monolayer graphene with previously prepared electrodes (see Materials and methods in the supporting information for details). The characteristic peaks of 10 nm pentacene emerge exclusively at 1155 and 1178 cm^−1^, whereas 5 nm C_60_ is characterized by the A_g_(2) peak (1464 cm^−1^). Valid interface contact and a smooth surface will ensure the carrier transport efficiency of the device. Therefore, morphological characterization of pentacene by atomic force microscopy was performed, showing a relatively flat surface in Fig. 1c (top panel), and the intermediate layer C_60_ is characterized in Fig. 1c (bottom panel). The C_60_/graphene contact breaks the electron-hole occupation symmetry in intrinsic graphene and tunes the Fermi level of graphene. Owing to the C_60_/graphene contact interaction, we estimate that the Dirac point of graphene shift toward positive voltages, indicating hole injection from C_60_ to graphene (Fig. 1d).

Because of the limited absorption range of C_60_/pentacene with a cutoff point of ~700 nm (Fig. S1), the prepared phototransistor shows positive and negative alternating photocurrents under different wavelength (from 405 to 1550 nm) illumination while keeping *V*_sd_ and the power density constant (Fig. 1e). The negative photocurrents stem from C_60_/pentacene electron injection into graphene, and the graphene/C_60_ heterojunction determines the positive response^23^. The positive photocurrents in the infrared region and negative photocurrents in the visible region suggest opposite light doping effects that modulate the graphene channel. The distinct optical doping effects are utilized to realize inhibition or promotion of the built-in field in the vertical heterojunction, and we use them to modulate the original optical signal. (Fig. 1a, f, opposite response regions act as signal light and modulation light).

**Fig. 2:**
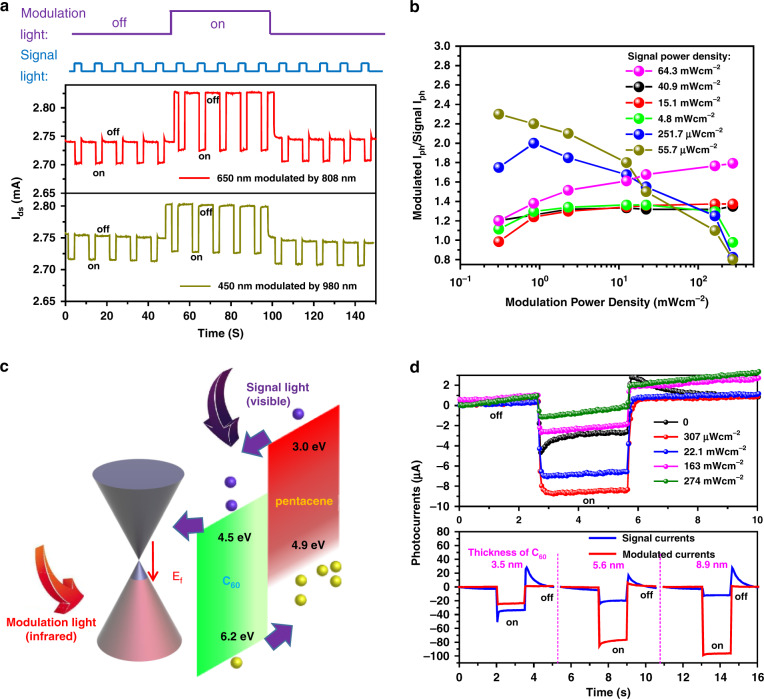
Photoresponse performance and visible response characteristics under infrared light modulation (5 nm thick C_60_ device). **a** Photocurrent enhancement of the visible 450 and 650 nm responses under infrared 980 nm and 808 light modulation, respectively (450 and 650 nm light power density = 20 mWcm^−2^, 808 and 980 nm modulation light power density = 20 mWcm^−2^, *V*_*ds*_ = 1 V). **b** Relationship among modulated *I*_*ph*_/signal *I*_*ph*_, modulation light power density and signal light power density. **c** Schematic diagram of visible region light (405–650 nm) working as signal light under the infrared light-modulation-induced carrier transfer process. The small purple arrow represents signal light-induced charge transfer. **d** The top panel shows the dynamic photocurrent response for low power density signal light (650 nm with a power of 23.4 μWcm^−2^) under different modulation light power densities (808 nm with 273 mWcm^−2^, 163 mWcm^−2^, 22.1 mWcm^−2^, and 307 μWcm^−2^). The bottom panel shows modulated *I*_*ph*_ (red line) and signal *I*_*ph*_ (blue line) as a function of C_60_ thickness (signal light: 650 nm with a power of 19.3 mW/cm^2^, modulation light: 808 nm with a power of 20 mW/cm^2^, *V*_*ds*_ = 1 V). Modulated *I*_*ph*_ is the photocurrent under modulation light control, and signal *I*_*ph*_ is the photocurrent of the intrinsic signal light

Contrary to the conventional scenario (in which positive and negative responses will inhibit each other), a peculiar situation occurs in our measurements. We excited a device with pulsed visible signal light (power density of 20 mW/cm^2^) by infrared optical on–off modulation, as shown in Fig. 2a. The optically modulated output signal current significantly rises nearly two- to threefold under 20 mW/cm^2^ infrared light and stably recovers after the modulation light is removed. In addition, different powers of the signal light or modulation light affect the trend of the output current. Figure 2b depicts the modulated dynamics of the output currents under various light power densities; *β* (*β* is an enhancement factor for easy comparison, which replaces modulated *I*_ph_/signal *I*_ph_) decreases sharply as the modulation light power density increases at relatively low signal light power densities (251.7 and 55.7 μW/cm^2^), while there is a slight rise in *β* at higher signal light power densities. As the bandgap of bilayer C_60_/pentacene forms a type-II heterojunction, the N-type C_60_ with a LUMO (HOMO) of 4.5 eV (6.2 eV) and P-type pentacene with a value of 3.0 eV (4.9 eV) (Fig. 2c)^36–38^ enable photogenerated electrons to propagate into graphene. The tuneable Schottky barrier derived from graphene/C_60_ affects the band alignment. The Fermi level of the bottom graphene is closely associated with the modulation light power density, which conclusively inhibits or facilitates carrier transfer. Therefore, the infrared modulation light causes P-type doping in graphene and directly enhances the built-in field of C_60_/pentacene (Fig. 2c), which allows electrons (signal light generated) in C_60_/pentacene to effectively be injected into graphene, resulting in a higher photocurrent (Fig. 2a). The appropriate power of the infrared light will cause P-doping of graphene and enable the signal light photogenerated electrons to be injected into graphene. However, excessive power of the infrared light can cause deep P-doping of graphene. Then, the signal light photogenerated electrons hardly induce a variation in the conductance due to the considerable quantity of carriers with the deep P-doping of graphene (Fig. 2d top panel)^39^. Therefore, matching the powers of the modulation light and signal light will lead to a larger enhancement (Fig. 2b).

By utilizing this trend, the photoresponsivity of the intrinsic visible signal light (15.4 μW/cm^2^) can be promoted from 3425 A/W to 7673 A/W under a proper modulation light power density of 307 μW/cm^2^ (Fig. 2d top panel). Under the same signal (650 nm with 19.3 mW/cm^2^) and modulation (808 nm with 22.1 mW/cm^2^) light, we examined the different thickness C_60_ devices (Fig. 2d bottom panel). As the thickness of C_60_ increases, *β* can reach 7.92, and a further explanation of the thickness variation effect will be introduced below. The device modulation performance also depends on the light modulation response, such as the inferior response when 1550 nm light acts as the modulation light, which has a relatively weak ability to realize enhanced modulation (Fig. S2).

**Fig. 3:**
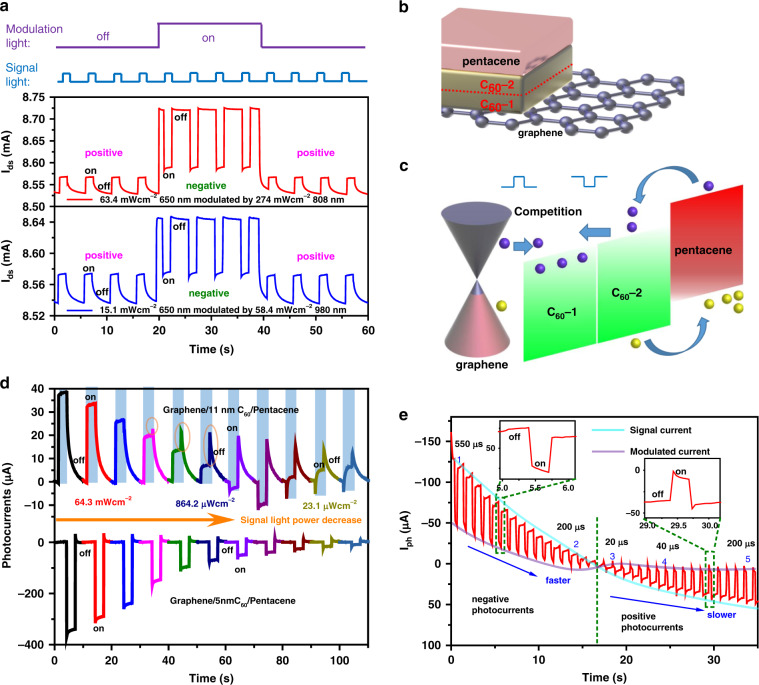
Control thick C_60_ (11.2 nm) device photoresponse characteristics and photocurrent reversal modulation. **a** Sign reversal modulation of the device. Dynamic photocurrent responses for 63.4 mWcm^−2^ 650 nm signal light under 307 μWcm^−2^ 808 nm infrared light switching modulation (top panel) and 15.1 mWcm^−2^ 650 nm signal light under 58.4 mWcm^−2^ 980 nm modulation (bottom panel). **b** Structure schematic of the intermediate transport layer exceeding 10 nm. **c** Photogenerated carrier transfer diagram of the competition between the two graphene/C_60_-1 (positive response) and C_60_-2/pentacene (negative response) systems. **d** Intrinsic photocurrent variations of the different C_60_ thickness (5 nm, bottom panel; 11 nm, top panel) devices under different 650 nm light power densities from 133.7 mWcm^−2^ to 7.7 μWcm^−2^ (each color represents a power density). The top panel shows a positive and negative alternating photocurrent, and the lower panel shows a large negative photocurrent decline as the light power density decreases. **e** Photoresponse at 450 nm (20 mWcm^−2^) under sustained 405 nm (37 mWcm^−2^) light modulation

To deeply clarify the light modulation mechanism mentioned above, we turn our focus to the thickness-dependent modulation in graphene/C_60_/pentacene. We fabricate an 11 nm C_60_ control device with the same pentacene thickness, which aims to force photogenerated electrons to be partly depleted at a distance (the effective exciton length is normally 5–10 nm)^39^. Compared with the previous 5 nm thick C_60_ light modulation measurements, which can only realize codirectional photocurrent modulation (Fig. 2a), the phototransistor with 650 nm signal light demonstrates obvious photocurrent polarity switching when modulated by infrared 808 or 980 nm light, as shown in Fig. 3a. This logical signal reversal by light modulation exhibits some similar features to optical inversion devices^40–43^. When the thickness of the intermediate transport layer exceeds or approaches the effective built-in electric field length, the entire system can be split into two parts, as displayed in Fig. 3b, c (the red dashed line in Fig. 3b): graphene/C_60_-1 and C_60_-2/pentacene. The C_60_-2/pentacene will exhibit a response for a radiation change of 400–700 nm, with its bandgap approaching 2 eV, and prompt electron movement toward graphene/C_60_-1 (as we note in Fig. 3c with an arrow). Owing to the formation of a CT state at the graphene/C_60_-1 interface (near-infrared region), appropriate energy photogenerated electrons can transfer into shallow impurities of C_60_ and be trapped in them. Photogenerated holes from C_60_ move into graphene under visible illumination. Therefore, both near-infrared and visible irradiation will produce positive photocurrents at the graphene/C_60_-1 interface, suggesting that the transmission direction of electrons points from graphene to C_60_ (as depicted in Fig. 3c with an arrow). Thus, if visible light irradiates the graphene/C_60_/pentacene heterojunction, then the opposite transfer directions derived from graphene/C_60_-1 and C_60_-2/pentacene will produce a response competition. In the thick C_60_ circumstance, the visible-light-triggered photogenerated carriers of C_60_-2/pentacene can hardly transfer into graphene because of depletion over the long distance, leading to a positive photocurrent in the preliminary response before infrared modulation (Fig. 3a). Infrared irradiation can induce P-doping of the graphene channel from the graphene/C_60_-1 interface. Therefore, the enhanced P-doping graphene owing to infrared light modulation promotes a built-in electric field for electron injection into graphene, and C_60_-2/pentacene will dominate the photoresponse, resulting in a negative photocurrent (Fig. 3a), which offers the chance to realize logical signal reversal. It is worth noting that the power of the signal light should be compatible with that of the modulation light, and more sign reversals of photocurrents for various modulation light power densities are shown in Fig. S3. The photocurrent reversal magnitude is also related to the modulation light power density. Hence, the competition is ascribed to the different interfaces, and the asymmetric carrier transfer path provides control of the dominant interface^44^. The infrared light modulation can regulate this competition and neutralize the thickness depletion, which is equivalent to the thin C_60_ device condition. This striking difference reveals electric field changes in the multilayer heterojunction system.

To further verify the inference proposed above, we explore power-dependent photocurrent measurements in two control groups without light modulation. As we know, there is no photocurrent reversal as a function of the input light power in most photoconductive devices. However, obviously distinct reversals of the time-resolved photocurrents between the devices with 5 nm and 11 nm thick C_60_ intermediate transport layers with signal light power variation are observed (Fig. 3d). From 133.7 mW/cm^2^ to 7.7 μW/cm^2^ light, the negative photocurrents imply that photogenerated electrons can effectively enter graphene owing to the C_60_-2/pentacene band alignment and proper thickness of intermediate transport layer C_60_ (Fig. 3d bottom panel), which accounts for codirectional photocurrents in light modulation (Fig. 2a). In contrast to the all negative photocurrents in the 5 nm C_60_ control device, we observe a two-times photocurrent reversal phenomenon in the 11 nm C_60_ device with decreasing power density (from 133.7 mW/cm^2^ to 7.7 μW/cm^2^) of the input 650 nm light (Fig. 3d top panel). As we can see, 650 nm illumination with a power density exceeding 864.2 μW/cm^2^ of the device (Fig. 3d top panel from the dark blue to black lines) results in positive photocurrents. These positive photocurrents are the combined result of two major parts of the response: (1) the photothermoelectric part and (2) photoelectric part. As deduced from this phenomenon, the thick C_60_ tends to contribute photothermoelectric currents, especially when we apply relatively high-power light. Under 650 nm light irradiation, the photogenerated excitons segregate and drift with the C_60_-2/pentacene band alignment. Then, the holes trapped in pentacene and a few electrons transfer into graphene owing to the depletion through 11 nm C_60_ (Fig. 3b), which is responsible for the relatively small negative response arising from the photoelectric part (from purple to dark purple lines in Fig. 3d top panel). We can even distinguish a pure negative response with an instantaneous sharp peak at the brink of the on–off switching moment (when the input power density is < 251.7 μW/cm^2^ in Fig. 3d top panel). Thus, there is still a negative response component in positive photocurrents (circled in Fig. 3d top panel). However, these sharp peaks decrease with increasing light power (from orange to dark blue lines in Fig. 3d top panel) and are ultimately submerged in the primary positive responses (from black to blue lines in Fig. 3d top panel) due to the saturation effect of photoelectric currents. By contrast, positive photocurrents exhibit remarkable growth in the corresponding power density range. Therefore, as the input light power increases, positive photothermoelectric currents will predominate in the response, causing a screening effect for the negative response. With degeneration of the photothermoelectric effect (when the 650 nm illumination power decreases to 251.7 μW/cm^2^), the built-in field of C_60_-2/pentacene leads to apparent electron injection (including drift and diffusion processes) into graphene, resulting in pure negative photocurrents (from violet to faint brown lines in Fig. 3d top panel). With the continuous reduction in the input light power, the concentration of electrons input into the graphene channel decreases to a low level, and few electrons can undergo the depletion or diffusion process through C_60_-1 and C_60_-2 (input power = 37.5 μW/cm^2^). Therefore, the negative photocurrents from C_60_-2/pentacene vanish, leaving only the sharp peaks (Fig. 3d), and the graphene/C_60_-1 interface boundary may dominate the photoresponse, leading to the positive photocurrents rising in magnitude. This fundamental power-dependent multiple state phenomenon in the photocurrent provides the possibility for photocurrent switching by light modulation.

We observed an intensive and slow negative drift in the variation in the photoconductance when stimulating the device by ultraviolet illumination without light modulation (the 405 nm intrinsic response depicted in Fig. S4). The complete positive and negative transition process under sustained 405 nm light modulation is presented in Fig. 3e. This figure shows that the photocurrent declines from negative 78.8 μA to 0 and rises from 0 to positive 36.3 μA, with the response time changing. This gradually varied negative response offers another sustained light modulation method. Similar to electrical gate modulation, this type of sustained 405 nm light modulation causes an N-type doping drift in the photocurrent measurements. Utilizing the power-dependent multiple states of the photocurrent caused by thick C_60_, this consecutive N-type doping can be used to easily tune intrinsic P-type graphene into N-type. The N-type doping drift of graphene o to ultraviolet light is attributed to oxygen chemisorption, and the ultraviolet light removes the chemisorbed oxygen on the surface of graphene. At present, the electrons from C_60_/pentacene transfer into N-type graphene, leading to positive photocurrents. We captured several transient response times measured by an oscilloscope (as shown in Fig. S5), which indicates a photoresponsivity-response time trade-off. The intrinsic response time can be modulated to as low as 20 μs for a weak positive photoresponse, as depicted in Fig. 3e. The intrinsic rise time (or response time) ranges from 20 to 550 μs, showing the τ-light modulation relationship in our device. The similar relationship can enable a parallel comparison with the τ–*V*_g_ relationship in the graphene/perylenetetracarboxylic dianhydride/pentacene device^24^. The relatively fast operation time (20 μs) at the cost of the photocurrent shows high-speed application potential in light modulation devices. Furthermore, the dynamic light-modulated photoresponsivity and response time suggest a thickness-dependent carrier transfer feature and potential for replacement of electrical modulation.

**Fig. 4:**
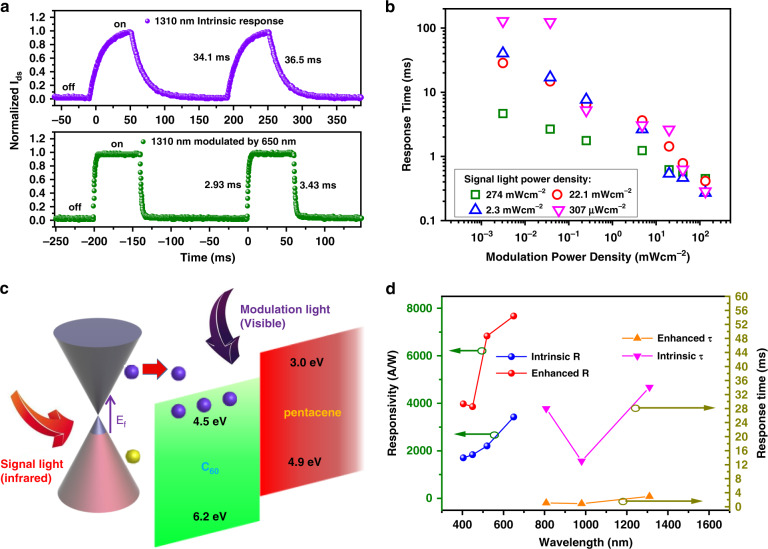
Photoelectric characterization of the infrared response under visible light switching modulation (5 nm thick C_60_ device). **a** Time-dependent photoresponse of the intrinsic infrared 1310 nm (20 mWcm^−2^) positive response and 1310 nm response under 650 nm 20 mWcm^−2^ light modulation measured by an oscilloscope. **b** Analysis of the response time of graphene/C_60_/pentacene devices at different signal (808 nm) and modulation (650 nm) light power densities, *V*_ds_ = 1 V. **c** Schematic diagram of infrared light (808–1550 nm) working as signal light under the visible-light-modulation-induced carrier transfer process; the small red arrow represents signal light-induced charge transfer. **d** Summary of the enhancement in the device performance of the bilayer/graphene heterojunction phototransistor in terms of the photoresponse and response time by light modulation (left panel modulation: signal light power density: 28.3 μWcm^−2^ (405 nm), 17.4 μWcm^−2^ (450 nm), 36.7 μWcm^−2^ (520 nm) 23.4 μWcm^−2^ (650 nm), and modulation light power density of nearly 300 μWcm^−2^; right panel modulation: signal and modulation light power densities of ~20 mWcm^−2^)

Finally, we carefully investigate the infrared response photocurrent variation with visible light modulation. Figure 4a plots the time-resolved variation characteristics of the infrared response, with a faster response time under visible light modulation. At the expense of the photocurrent, the rise and recovery times of the positive 1310 nm response (power density = 20 mW/cm^2^) can be reduced from 34.1 and 36.5 ms to 2.93 and 3.43 ms under 650 nm illumination with a power of 20 mW/cm^2^ at the cost of a low gain (G). As the variation in the Fermi level is associated with the power density of the modulation light, the distinct doping levels of graphene affect the band bending and thus the transmission, which is closely related to the response time. Therefore, we present the power-dependent response time curves in Fig. 4b, which indicate an operable tuneable bandwidth from 10 to 3 × 10^3^ Hz. All curves demonstrate an improved response time as the modulation light power density increases, and the response time can be promoted ~10^3^ times when 307 μW/cm^2^ 808 nm signal light is modulated by 650 nm light with a power of 133.7 mW/cm^2^. Distinct from the visible response modulated by infrared light, we found complete inhibition of the photocurrent in different light modulation bands (Fig. S6a, b). The C_60_/pentacene bilayer generates the majority of electrons injected into graphene, resulting in N-type doping under visible light illumination. The intrinsic positive photocurrents can be modulated to produce smaller photocurrents owing to the built-in electric field of graphene/C_60_ diminishing as graphene N-type doping is enhanced (see Fig. 4c). To obtain more details of the visible light modulation, we also perform photocurrent dynamic measurements under different incident modulation light power densities (see Fig. S6c); as the modulation light power density decreases, all curves shows a decline in the photocurrent modulation result. Compared with the infrared light modulation, no direction variation but a decline in the photocurrent is observed even at low power densities of the signal and modulation light (as shown in Fig. S7).

## Discussion

The light modulation method mentioned above involves mutual modulation between two light wavelength regimes (except for the special circumstance of 405 nm modulation with removal of chemisorbed oxygen). Therefore, this graphene/C_60_/pentacene structure splits the detection range into two regions, 405–650 and 808–1550 nm, owing to the bidirectional response. The upper layer material determines the positive and negative response transition points, which can affect the distinct light modulation regions. To exclude some accidental factors, the photoresponse characteristics when the dynamics are modulated in one region (both the signal and modulation light are in one region) are investigated in Fig. S8, which exhibits a normal saturation relation between them. Different from the traditional hybrid modulation phototransistor using one gate light or source light beam to tune signals, our all-light-input phototransistor with a tuneable dynamic response feature under light modulation demonstrates that light modulation can replace most of the function of electrical modulation by a gate and even trigger multiple state competition in the vertical direction. The photocurrent polarity variation phenomenon with the substitution of electrical modulation paves the way for developing polarity-controlled all-optical-input devices^7^.

The high photoresponsivity and low response time of the 5 nm C_60_/10 nm pentacene device can be achieved through our two distinct light modulations, where the two important parameters are enhanced in different situations, as summarized in Fig. 4d (the left panel shows modulation by infrared light, and the right panel shows modulation by visible light). The promotion of photocurrents via suitable infrared modulation light power ensures efficient transport of carriers and avoids a saturation effect at the cost of the recovery time in the visible region. The visible light N-doping modulation improves the response and recovery times at the sacrifice of the photocurrent. Moreover, the asymmetric amplitude and response time of the logical photocurrent reversals may affect back-end signal acquisition. However, it is advantageous that infrared modulation light can act as a switch to turn on or off the logical signal reversal function, and simultaneously, all the input signals and modulations were based on light.

In summary, we demonstrated two distinct light modulations in thin and thick C_60_ bilayer phototransistors (graphene/C_60_/pentacene). The thick C_60_ layer device exhibits a more distinct modulation phenomenon as the thickness of the intermediate transport layer increases, realizing logical switching via light modulation, which shows great potential for fabricating optical polarity modulation devices. Continuous violet illumination enables us to observe a repeatable positive-negative alternating process and an intrinsic visible response τ tuneable down to ~20 μs. Moreover, the visible light modulation provides a tuneable bandwidth from 10 to 3 × 10^3^ Hz for adjusting infrared photocurrents. The power-related tuneable photocurrent phenomenon provides a new effective light modulation method for enhanced response, where the photoresponse is increased from 3425 A/W up to 7673 A/W. These light modulation results not only reveal the carrier transport mechanism in bidirectional phototransistors but also present the advantage and mechanism of light modulation, which provides a reliable method for future development of more innovative and functional optoelectronic devices.

## Materials and methods

### Device fabrication and characterization

Single-layer CVD graphene was transferred to an n+ Si/SiO_2_ substrate (SiO_2_ 285 nm) by a solution method. The CVD graphene was purchased from Nanjing MKNANO Technology Inc. The 5 nm C_60_ (at 430 °C) deposition rate was 0.02 nm/s (vacuum = 10^−5^ Torr). Then, 10 nm thick pentacene (at 155 °C) was vapor-deposited onto 5 nm C_60_ at a starting deposition rate of 0.02 nm/s, which was gradually increased to 0.04 nm/s. All film thicknesses mentioned above were determined by a SQC-310 deposition controller. The pentacene material, rated at over 98% purity, was purchased from MREDA Technology Inc. made in the USA. The C_60_ material, rated at over 99.5% purity, was purchased from Polymer Xi’an Baotelai Light Technology Inc.

### Light-modulation measurement methods

We used a response laser beam and a modulation laser beam to investigate light modulation characteristics. The transfer characteristics and light modulation optoelectronic characteristics data were measured by a Keithley 2636b source meter analyzer and a PDA FS-Pro semiconductor analyzer with a probe station. Highly time-resolved photocurrent signals under light modulation were obtained by an oscilloscope with a pulse source, which were amplified and detected using the current amplifier (Stanford SR570) technique.

The absorption data of the graphene/C_60_/pentacene and C_60_/pentacene control groups were collected by a UV2600, and Raman spectra measurements were performed by a confocal microprobe Raman spectrometer (RENISHAW inVia Raman Microscope) under the illumination of a 514 nm helium-neon laser with a power of 2 mW.

## Supplementary information

supporting materials
