# Supplementary material for: Light-modulated vertical heterojunction phototransistors with distinct logical photocurrents
Source: Light Sci Appl. 2020 Sep 23;9:167. doi: 10.1038/s41377-020-00406-4 (PMC7509774; doi:10.1038/s41377-020-00406-4)
Supplement: Supplementary file 1 — supporting materials [file 41377_2020_406_MOESM1_ESM.docx]

Supporting Information for Light-Modulated Vertical Heterojunction Phototransistors with Distinct Logical Photocurrents

Jiayue Han,^†^ Meiyu He,^†^ Ming Yang,^†^ Qi Han,^†^ Fang Wang,^‡^ Fang Zhong,^‡^ Mengjian Xu,^‡^ Qing Li^‖^, He Zhu^‖^, Chongxin Shan,^§^ Weida Hu,*^,‡,‖^ Xiaoqing Chen, ^○^ Xinran Wang,*^,○^Jun Gou, ^†,ξ^ Zhiming Wu, ^†,ξ^ and Jun Wang*^,†,ξ^

†School of Optoelectronic Science and Engineering, University of Electronic Science and Technology of China, Chengdu 610054, China

‡State Key Laboratory of Infrared Physics, Shanghai Institute of Technical Physics, Chinese Academy of Science, 500 Yutian Road, Shanghai 200083, China

§Henan Key Laboratory of Diamond Optoelectronic Materials and Devices, School of Physics and Engineering, Zhengzhou University, Zhengzhou 450001, China

○National Laboratory of Solid State Microstructures, School of Electronic Science and Engineering, and Collaborative Innovation Center of Advanced Microstructures, Nanjing University, Nanjing 210093, China

ξState Key Laboratory of Electronic Thin Films and Integrated Devices, University of Electronic Science and Technology of China, Chengdu 610054, China

*^‖^*Hangzhou Institute for Advanced Study, University of Chinese Academy of Sciences, Hangzhou 310024, China

* E-mail: [wdhu@mail.sitp.ac.cn (Wei](mailto:wdhu@mail.sitp.ac.cn%20(Wei)da Hu)

* E-mail: [xrwang@nju.edu.cn](mailto:xrwang@nju.edu.cn) (Xinran Wang)

* E-mail: [wjun@uestc.edu.cn](mailto:wjun@uestc.edu.cn) (Jun Wang)

1. The absorption spectrum


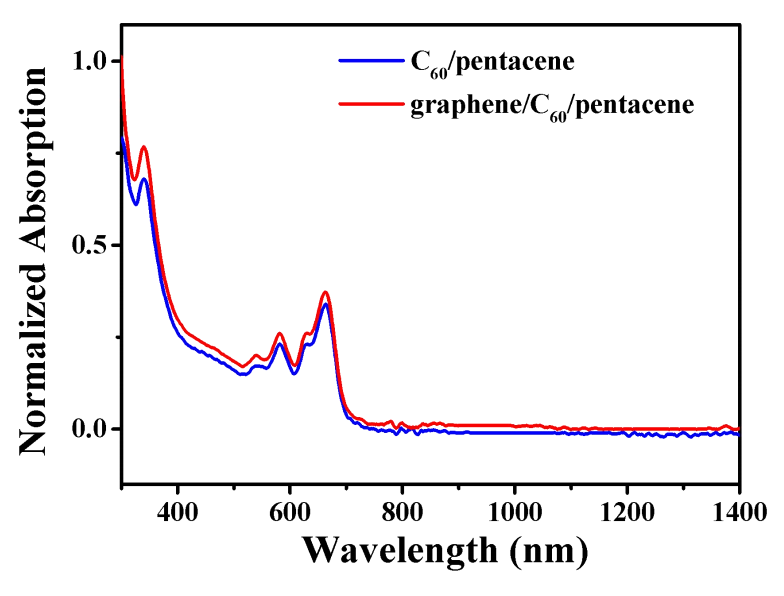


Fig. S1. The absorption spectrum of graphene/C_60_/pentacene and C_60_/pentacene, respectively.

2. The optical modulation characterization, 405 nm response modulated by 1550 nm.


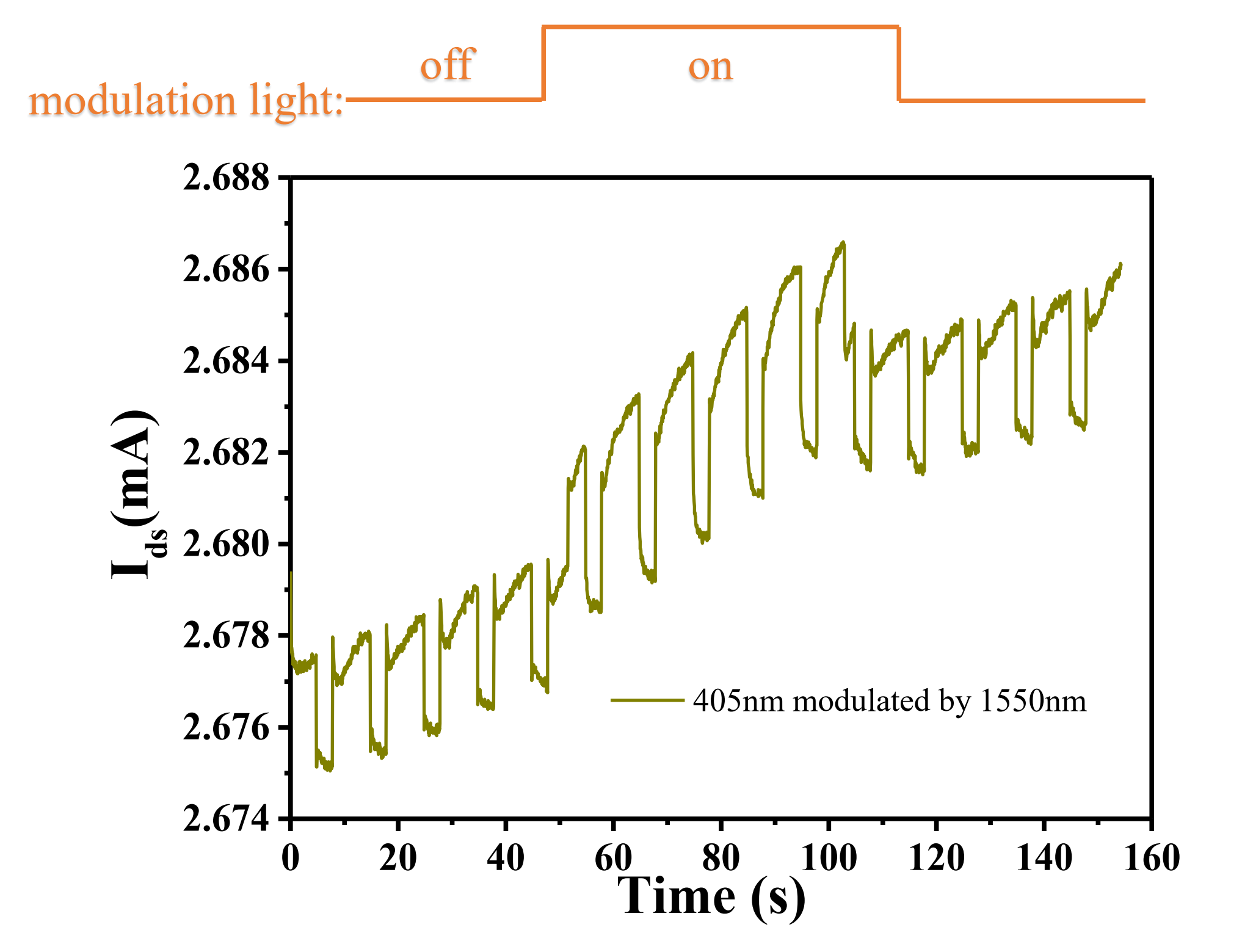


Fig. S2. The responsivity of graphene/C_60_/pentacene in 405 nm response (405 nm laser power density=32.7 μW cm^-2^) under 550 nm laser illumination modulation (1550 nm laser power density=20.34 mW cm^-2^).

The structure and photoresponse characterization of the device’s intermediate transport layer exceeding 10 nm


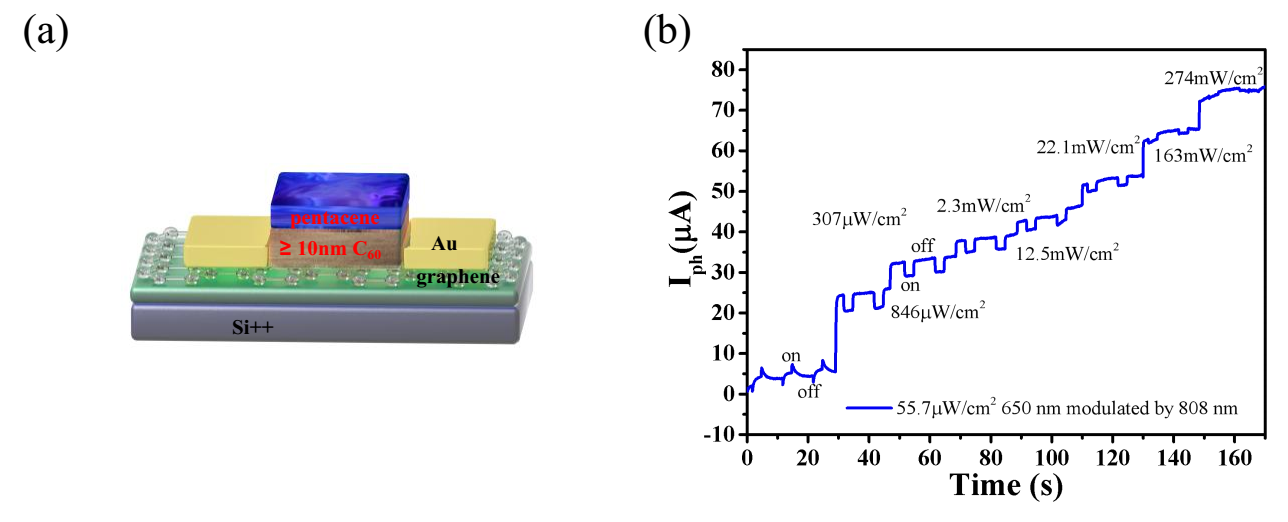
Fig. S3. (a)The structure of thick C_60_ device. (b) The sign reversal photocurrent response in thicker C_60_ device of the low power density (55.7 μWcm^-2^) as the function of different modulation power density (808 nm).

4. The 405 nm intrinsic response of the thick (11.2 nm) C60 device.

Fig. S4. The 405 nm intrinsic response of the thicker C_60_ device.

5. The 450 nm response under the sustained 405 nm optical modulation of the thick (11.2 nm) C_60_ device.


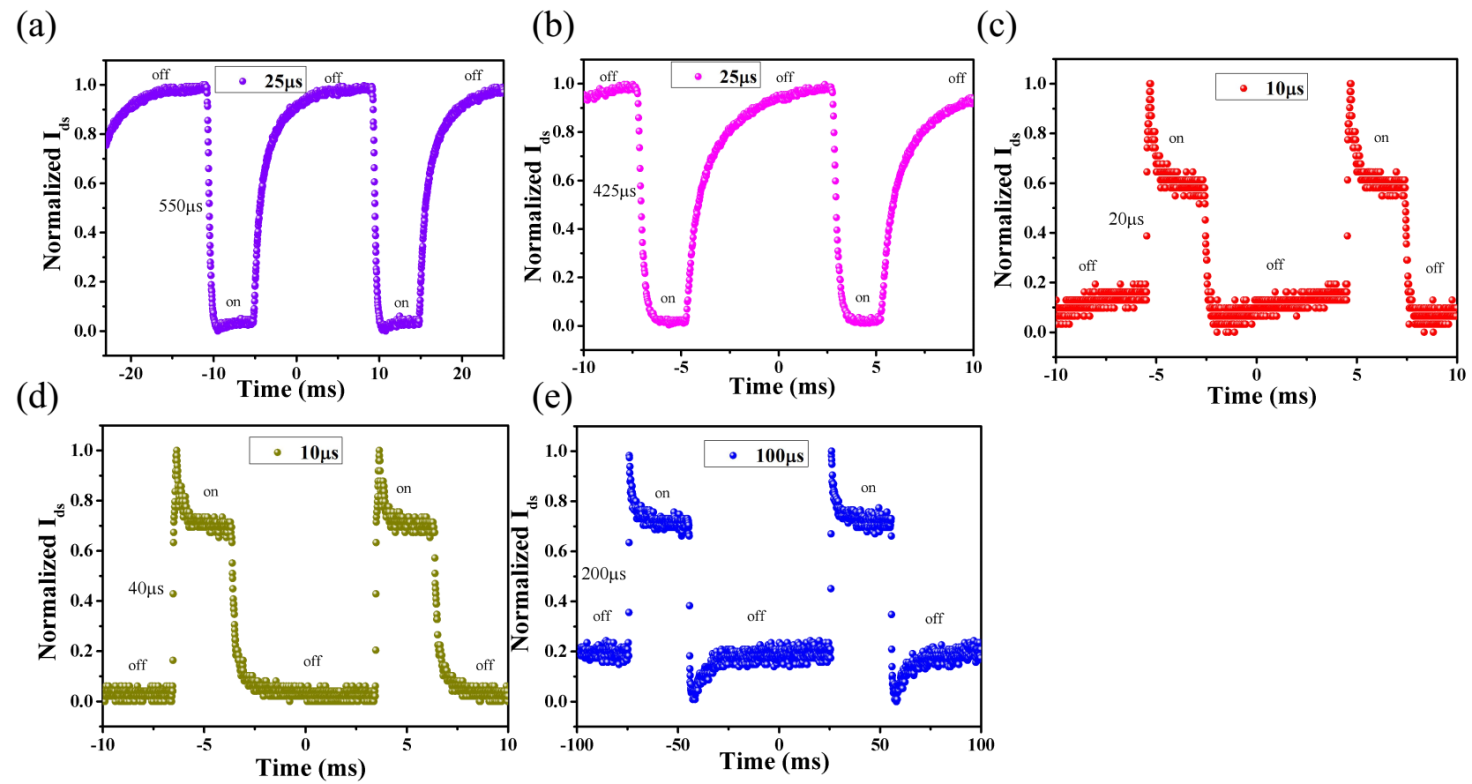


Fig. S5. (a,b,c,d,e) Five temporal 450 nm photocurrents response represent the Fig. 4d corresponding point 1,2,3,4 and 5 under the sustained 405 nm optical modulation.

6. The optical modulation characterization.


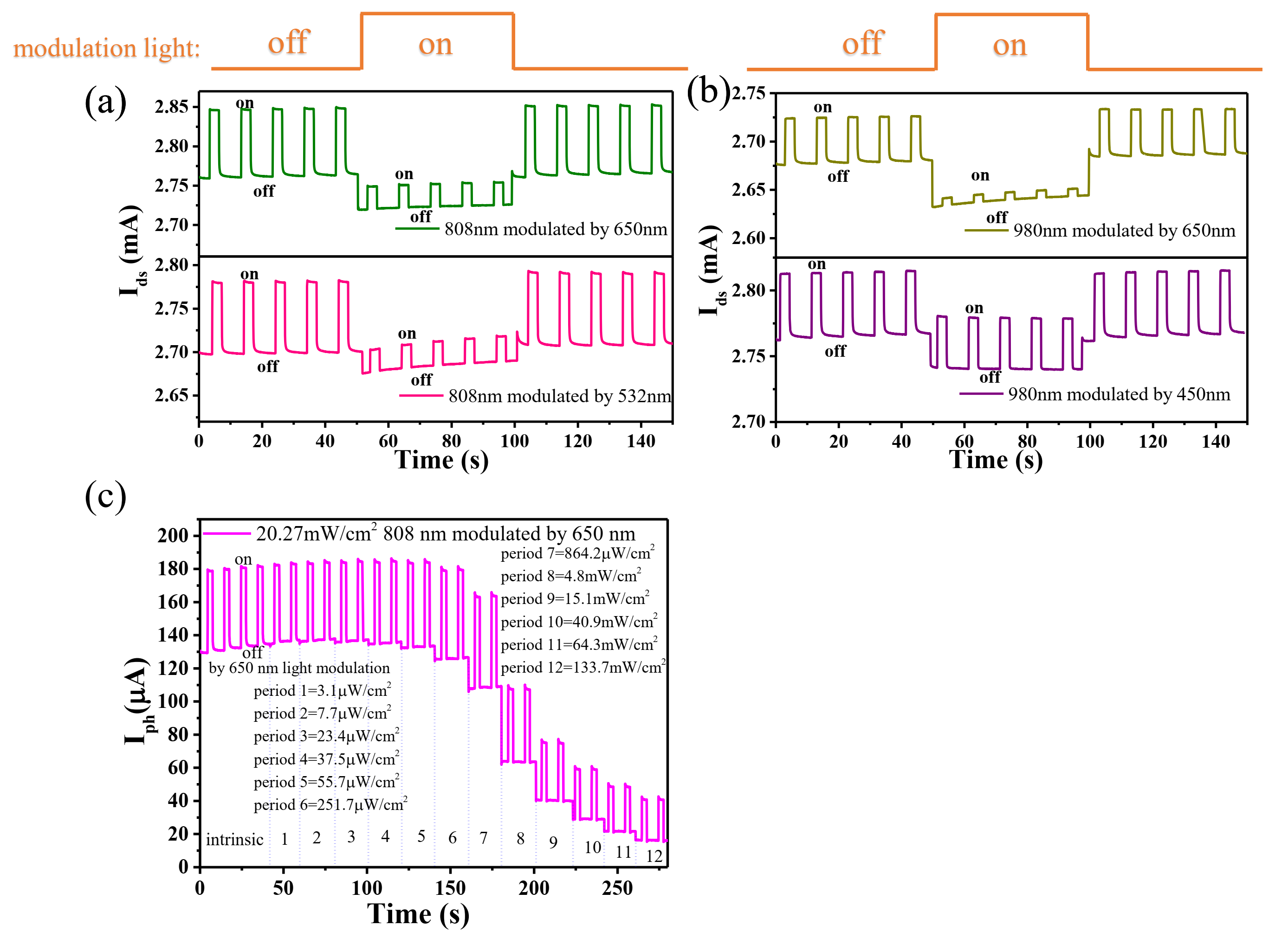


Fig. S6. (a) The I-t characteristics of infrared 808 nm response under the light 532 and 650 nm switch modulation, (808 nm response power density=83.1 mWcm^-2^ and 532, 650 nm modulation power density=20 mWcm^-2^, V_ds_=1V). (b) The I-t characteristics of infrared 980 nm response under the gate light 450 and 650 nm switch modulation, (808 nm response power density=83.1 mWcm^-2^ and 450, 650 nm modulation power density=20 mWcm^-2^, V_ds_=1V). (c) The infrared 808 nm response modulation by distinct power density of 650 nm light.

7. The I-t characteristics of infrared 808 nm response under the light 650 nm switch modulation.


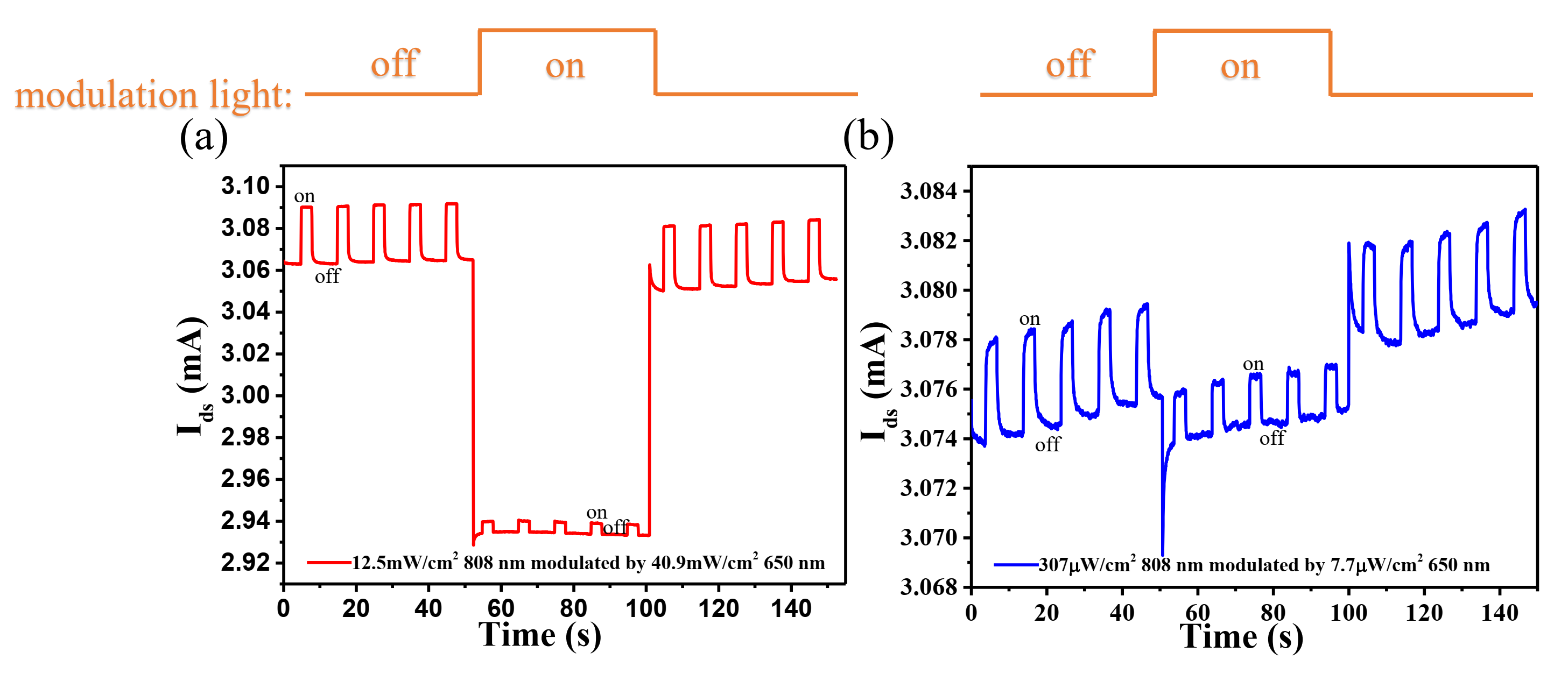


Fig. S7. (a) The relatively high power density infrared 808 nm response modulation by high power density of 650 nm light. (b) The relatively low power density infrared 808 nm response modulation by low power density of 650 nm light.

8. The competing saturated photoresponse characterization in both positive response or negative response region.


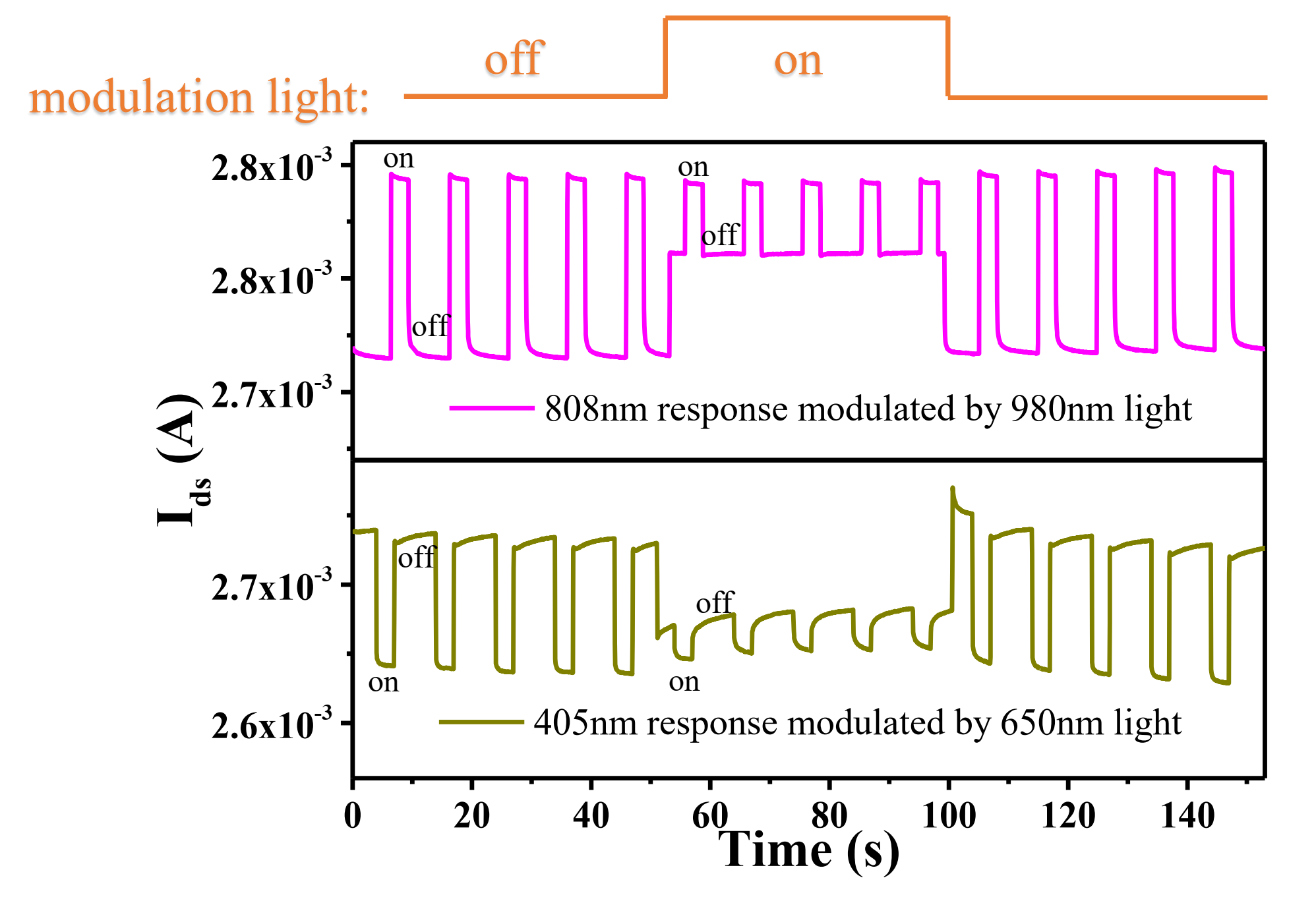


Fig. S8. optical modulation responsivity of graphene/C_60_/pentacene in both positive response (808-1550 nm) or negative response (405-650 nm) region.
